# During Stably Suppressive Antiretroviral Therapy Integrated HIV-1 DNA Load in Peripheral Blood is Associated with the Frequency of CD8 Cells Expressing HLA-DR/DP/DQ

**DOI:** 10.1016/j.ebiom.2015.07.025

**Published:** 2015-07-21

**Authors:** Alessandra Ruggiero, Ward De Spiegelaere, Alessandro Cozzi-Lepri, Maja Kiselinova, Georgios Pollakis, Apostolos Beloukas, Linos Vandekerckhove, Matthew Strain, Douglas Richman, Andrew Phillips, Anna Maria Geretti, ERAS Study Group, Paola Vitiello, Nicola Mackie, Jonathan Ainsworth, Anele Waters, Frank Post, Simon Edwards, Julie Fox

**Affiliations:** 1 Royal Free Hospital, London and The Royal Liverpool Hospital, Liverpool, United Kingdom; 2 St Mary's Hospital, London, United Kingdom; 3 North Middlesex Hospital, London, United Kingdom; 4 King's College Hospital, London, United Kingdom; 5 University College Hospital, London, United Kingdom; 6 St Thomas' Hospital, London, United Kingdom; a Department of Clinical Infection, Microbiology and Immunology (CIMI), Institute of Infection and Global Health (IGH), University of Liverpool, 8 West Derby Street, Liverpool L697BE, United Kingdom; b HIV Translational Research Unit, Department of Internal Medicine, Ghent University and University Hospital Ghent, De Pintelaan 1859000, Ghent, Belgium; c Department of Infection and Population Health, University College London, Royal Free Campus, Rowland Hill Street, London, NW32PF, United Kingdom; d VA San Diego Healthcare System and Center for AIDS Research, University of California San Diego, La Jolla, CA 92093, United States

**Keywords:** HIV-1, Human Immunodeficiency Virus type 1, HIV-1 VL, HIV-1 viral load, ART, Anti-retroviral therapy, HIC, HIV-1 controllers, NNRTI, Non-nucleoside reverse-transcriptase inhibitors, NRTI, nucleoside/nucleotide reverse transcriptase inhibitors, VLS, Viral Load Suppression, PBMCs, Peripheral blood mononuclear cells, 2-LTR, 2-long terminal repeats, sCD14, soluble CD14, HLA, Human Leukocyte Antigen, CMV, cytomegalovirus virus, EBV, Epstein-Bar virus, NIHR, National Institute for Health Research, CRN, Clinical Research Network, WHO, World Health Organisation, ELISA, enzyme-linked immune-enzymatic assay, PFA, paraformaldehyde, LPS, lipopolysaccharide, PCR, Polymerase chain reaction, Suppression, Reservoir, Persistence, Integration, Activation

## Abstract

**Background:**

Characterising the correlates of HIV persistence improves understanding of disease pathogenesis and guides the design of curative strategies. This study investigated factors associated with integrated HIV-1 DNA load during consistently suppressive first-line antiretroviral therapy (ART).

**Method:**

Total, integrated, and 2-long terminal repeats (LTR) circular HIV-1 DNA, residual plasma HIV-1 RNA, T-cell activation markers, and soluble CD14 (sCD14) were measured in peripheral blood of 50 patients that had received 1–14 years of efavirenz-based or nevirapine-based therapy.

**Results:**

Integrated HIV-1 DNA load (per 10^6^ peripheral blood mononuclear cells) was median 1.9 log_10_ copies (interquartile range 1.7–2.2) and showed a mean difference of 0.2 log_10_ copies per 10 years of suppressive ART (95% confidence interval − 0.2, 0.6; p = 0.28). It was positively correlated with total HIV-1 DNA load and frequency of CD8^+^HLA-DR/DP/DQ^+^ cells, and was also higher in subjects with higher sCD14 levels, but showed no correlation with levels of 2-LTR circular HIV-1 DNA and residual plasma HIV-1 RNA, or the frequency of CD4^+^CD38^+^ and CD8^+^CD38^+^ cells. Adjusting for pre-ART viral load, duration of suppressive ART, CD4 cell counts, residual plasma HIV-1 RNA levels, and sCD14 levels, integrated HIV-1 DNA load was mean 0.5 log_10_ copies higher for each 50% higher frequency of CD8^+^HLA-DR/DP/DQ^+^ cells (95% confidence interval 0.2, 0.9; p = 0.01).

**Conclusions:**

The observed positive association between integrated HIV-1 DNA load and frequency of CD8^+^DR/DP/DQ^+^ cells indicates that a close correlation between HIV persistence and immune activation continues during consistently suppressive therapy. The inducers of the distinct activation profile warrant further investigation.

## Highlights

• Data from a homogenously treated population with consistent virological suppression
• Integrated HIV-1 DNA load did not vary significantly by duration of therapy
• Integrated HIV-1 DNA load was not associated with markers of recent virus replication
• Integrated HIV-1 DNA load and CD8+HLA-DR/DP/DQ+ frequency were positively associated
• Subjects with top quartile integrated HIV-1 DNA load showed high sCD14 levels

Integrated HIV-1 DNA load remains constant in the peripheral blood of individuals receiving long-term suppressive antiretroviral therapy (ART). However, the mechanisms preventing decay of the reservoir remain unclear. We studied a cross-sectional population, defined by the duration of suppressive ART. Integrated HIV-1 DNA load did not differ significantly according to the duration of suppressive ART, and showed no association with direct or indirect markers of ongoing virus replication. Rather, there was an independent, positive association between integrated HIV-1 DNA load and the frequency of CD8 cells expressing the activation marker HLA-DR/DP/DQ. These cells appear to have important regulatory and effector function. Our findings add to growing evidence that immune activation sustains the HIV-1 reservoir during long-term suppressive ART.

## 1 Introduction

Early after transmission, HIV establishes a reservoir of replication-competent integrated provirus that resides predominantly within memory CD4 T cells, is unresponsive to antiretroviral therapy (ART), and rapidly fuels resumption of virus replication upon treatment discontinuation (Ruelas and Greene, 2013; Soriano-Sarabia et al., 2014; Siliciano et al., 2003). The mechanisms underlying HIV persistence have not been fully elucidated and proposed models are not mutually exclusive. Continuous replenishment of the reservoir may occur through ongoing virus production (Hatano et al., 2013; Buzon et al., 2010), perhaps in sites where ART penetration or activity is suboptimal (Fletcher et al., 2014). In the absence of virus production, maintenance of the reservoir can also occur through proliferation of latently infected CD4 T-cells, as a possibly key determinant of HIV persistence during ART (Maldarelli et al., 2014; Josefsson et al., 2013; Chomont et al., 2009; Wagner et al., 2014).

HIV replication causes chronic immune activation and inflammation, typically accompanied by an expansion of CD8 T-cells, which improve but rarely resolve with ART (Klatt et al., 2013). Persistent HIV production or partial antigenic expression may drive ongoing immune activation in treated patients. In turn, immune activation can promote T-cell proliferation, HIV transcription, and virus production (Klatt et al., 2013). Furthermore, early in the course of the infection, HIV-induced gut damage allows translocation of gastrointestinal microbial products into the systemic circulation, and the resulting immune activation causes further gut damage, establishing a self-perpetuating cycle that can become independent of virus production (Shan and Siliciano, 2014). Other persistent infections, for example with cytomegalovirus (CMV), have also been linked with ongoing immune activation despite suppressive ART (Gianella et al., 2014).

There are inconsistent data on how markers of immune activation correlate with parameters of HIV persistence during long-term suppressive therapy, possibly a consequence of heterogeneous study populations and measures of HIV persistence, but also a reflection of complex bilateral interactions (Hatano et al., 2013; Chun et al., 2011; Cockerham et al., 2014). There remains a need to characterise this relationship further in order to improve our understanding of HIV pathogenesis and design improved curative strategies. The aim of this study was to investigate factors associated with the levels of integrated HIV-1 DNA measured in peripheral blood during stably suppressive ART, including the relationship with markers of immune activation. Efforts were taken to minimise heterogeneity of the study population, by exclusively selecting patients who started first-line ART with two nucleoside/nucleotide reverse transcriptase inhibitors (NRTIs) plus one non-nucleoside reverse transcriptase inhibitor (NNRTI), achieved a plasma HIV-1 RNA (“viral”) load < 50 copies/ml within six months of starting ART, and subsequently showed consistent viral load suppression < 50 copies/ml over up to 14 years of continuous treatment on the same NNRTI.

## 2 Methods

### 2.1 Study population

Study subjects were recruited at clinical centres across the United Kingdom (see study group). Eligible patients had started first-line ART with two NRTIs plus efavirenz or nevirapine, achieved viral load suppression < 50 copies/ml within six months of starting therapy, and subsequently showed all viral load measurements < 50 copies/ml whilst undergoing ≥ 2 viral load measurements per year, without transient elevations above 50 copies/ml or treatment interruptions. Changes of the initial NNRTI were not allowed. Changes of the initial NRTI (e.g., for toxicity) were allowed provided they were not associated with a treatment interruption or viral load rebound > 50 copies/ml. Recruitment was stratified by duration of ART to range from 1 to over 10 years. The study was approved by National Research Ethics Service (London-Dulwich) and was included in the National Institute for Health Research Clinical Research Network (NIHR CRN) Portfolio. All patients provided written informed consent.

### 2.2 Quantification of residual HIV-1 RNA in plasma

Plasma HIV-1 RNA below 50 copies/ml was quantified using a modified version of the Abbott RealTime HIV-1 assay (Maidenhead, UK), following ultracentrifugation of 8 ml of plasma at 215,000 g for 45′ at 4 °C, and resuspension of the pellet in 1 ml of basematrix (SeraCare, USA). Each run included negative, low positive, and high positive controls. Assay sensitivity was determined by spiking HIV-negative plasma with four dilutions of the World Health Organisation (WHO) 3rd International Standard for HIV-1 RNA in triplicate (NIBSC code:10/152, Hertfordshire, UK). The assay 50% and 95% detection rates for 8 ml input were 1 and 3 HIV-1 RNA copies/ml, respectively.

### 2.3 HIV-1 subtyping

The HIV-1 subtype was determined using polymerase sequences obtained from HIV-1 DNA recovered from PBMC, as previously described (Geretti et al., 2014).

### 2.4 Quantification of total, integrated and 2-LTR circular HIV-1 DNA in peripheral blood mononuclear cells

Total HIV-1 DNA load in peripheral blood mononuclear cells (PBMC) was determined by a quantitative real-time PCR targeting a conserved LTR region as previously described (Geretti et al., 2013). The assay 50% and 95% detection rates were 20 and 40 HIV-1 DNA copies/10^6^ PBMC, respectively. Integrated HIV-1 DNA was quantified by repetitive-sampling Alu-PCR as previously described (De Spiegelaere et al., 2014) using the primers shown in Supplementary Table 1. Briefly, 42 replicate Alu-HIV-1 PCR amplifications were performed with gag reverse HIV-1 primer and Alu forward primer for integrated HIV-1 DNA, and 32 replicates with only HIV-1 gag primers for unintegrated HIV-1 DNA. After amplification, the first PCR product was subjected to a nested quantitative PCR targeting the HIV-1 LTR RU5 region. The integrated HIV-1 DNA copy number was calculated according to Poisson's principles with error estimation including the Wilson method (www.integratedhivpcr.ugent.be). Four Alu-HIV positive wells were required as output to allow reliable quantification. 2-LTR circular HIV-1 DNA levels were measured by droplet digital PCR as previously described (Strain et al., 2013); the lower limit of detection was median 5 copies/10^6^ PBMC (interquartile range, IQR 4–6).

### 2.5 Markers of immune activation

Cellular markers were measured by staining freshly isolated PBMC with PE or FITC labelled antibodies against CD4 plus either CD26, CD38, or CD69, and against CD8 plus either CD38 or HLA-DR/DP/DQ (Serotec, Oxford, UK; Becton Dickinson, NJ, USA). Cell suspensions were fixed with 1% paraformaldehyde (PFA) prior to acquisition using FACScan and analysis by CellQuest software version 3.3 (Becton Dickinson NJ, USA). Soluble CD14 (sCD14) as a marker of bacterial lipopolysaccharide (LPS)-induced monocyte/macrophage activation was measured in plasma by enzyme-linked immune-enzymatic assay (ELISA) (R&D System sCD14 ELISA, Abington, UK).

### 2.6 Detection of cytomegalovirus and Epstein–Barr virus DNA

Plasma was tested for CMV and Epstein–Barr Virus (EBV) DNA with a real-time PCR assay targeting the viral UL123/UL55 and p143 regions, respectively. The lower limits of detection were 50 IU/ml for CMV DNA and 100 copies/ml for EBV DNA.

### 2.7 Statistical analysis

Duration of suppressive ART was defined as the length of time following the first viral load measurement < 50 copies/ml recorded after starting ART. The characteristics of the study population stratified into two groups based upon duration of suppressive ART, integrated HIV-1 DNA load, or frequency of CD8^+^HLA-DR/DP/DQ^+^ cells were compared using non-parametric Wilcoxon and Mann–Witney tests for continuous variables, and the Fisher's exact test for categorical variables. Where the analyses required variables to be expressed in median levels, undetectable HIV-1 RNA results were given an arbitrary midpoint value between zero and the assay 95% detection rate (= 1.5 copies/ml), whereas undetectable 2-LTR circular HIV-1 DNA results were assigned a midpoint value between zero and the assay median lower limit of detection (= 2.5 copies/10^6^ PBMC). Mean differences (with 95% confidence interval, CI) in measured residual plasma HIV-1 RNA, 2-LTR circular HIV-1 DNA, total and integrated HIV-1 DNA, sCD14, and frequency of activated CD4 and CD8 cells over 10 years of suppressive ART were analysed by univariate linear regression analysis after log transformation of the variables. The correlation between integrated HIV-1 DNA load or frequency of CD8^+^HLA-DR/DP/DQ^+^ cells and other measured parameters was explored by the Spearman's test. The association between integrated HIV-1 DNA load and other variables was characterised further by univariate and multivariable linear regression modelling. A ‘best subset’ approach and the Mallow Cp test were used for the selection of variables to be included in the multivariable model. All available variables were initially considered for inclusion, with the exception of gender and NNRTI use (due to predominance of males and efavirenz use: 40/50, 80% for both variables) and total HIV-1 DNA load (due to strong co-linearity with integrated HIV-1 DNA load). Pre-ART viral load (Cp value 3.5), duration of suppressive ART (3.6), residual plasma HIV-1 RNA load (3.6), frequency of CD8^+^HLA-DR/DP/DQ^+^ cells (0.4), and sCD14 levels (1.7) were identified for inclusion in the multivariate model. The CD4 cell count was added to the multivariable model as a possible confounding factor. In a second multivariable model, nadir CD4 count was included instead of pre-ART viral load. A sensitivity analysis was also performed replacing integrated with total HIV-1 DNA as the outcome variable in the same two models. The analyses were performed with SAS 9.4.

## 3 Results

### 3.1 Study population

The cohort comprised 50 patients who at the time of sampling were receiving two NRTIs plus either efavirenz (80%) or nevirapine and had a median duration of viral load suppression < 50 copies/ml of 6.4 years (Table 1). Patients with duration of suppressive ART above the median of 6.4 years were older and had a lower nadir CD4 cell count, a marginally higher current CD4 cell count, and lower frequencies of CD4 and CD8 cells expressing CD38 relative to subjects with shorter duration. They were also more likely to have experienced changes in the composition of the NRTI backbone after first starting ART. Overall 25/50 patients (50%) changed one or more component of the NRTI backbone, with median 1 drug change per subject (IQR 0–1). At the time of sampling, NRTI backbones were tenofovir/emtricitabine (37/50, 74%), abacavir/lamivudine (10/50, 20%), or zidovudine/lamivudine (3/50, 6%). Residual plasma HIV-1 RNA was detected in 29/50 patients (58%) at levels ranging between 1 and 35 copies/ml, whereas 2-LTR circular HIV-1 DNA was detected in 16/50 patients (32%) at levels ranging between 5 and 35 copies/10^6^ PBMC. All subjects had detectable HIV-1 DNA, with total and integrated HIV-1 DNA levels of median 2.6 and 1.9 log_10_ copies/10^6^ PBMC, respectively. None of the patients had detectable CMV DNA in plasma, whereas 3/50 patients (6%) had detectable EBV DNA.

Differences in measured parameters per 10 years of suppressive ART were determined by linear regression analysis of data obtained from the cross-sectional sampling (Table 2). Longer duration of suppressive ART was associated with higher CD4 cell counts and lower levels of CD38 expression on CD4 and CD8 cells. There were also lower 2-LTR circular HIV-1 DNA levels and a trend for lower residual HIV-1 RNA levels. A sub-analysis compared subjects with integrated HIV-1 DNA load either in the lowest quartile (< 1.7 log_10_ copies/10^6^ PBMC) or the highest quartile (> 2.2 log_10_ copies/10^6^ PBMC) (Table 3). Subsets in the lowest quartile showed significant lower levels of total HIV-1 DNA and sCD14, and lower frequency of CD8^+^HLA-DR/DP/DQ^+^ cells.

### 3.2 Factors associated with integrated HIV-1 DNA load

**Fig. 1:**
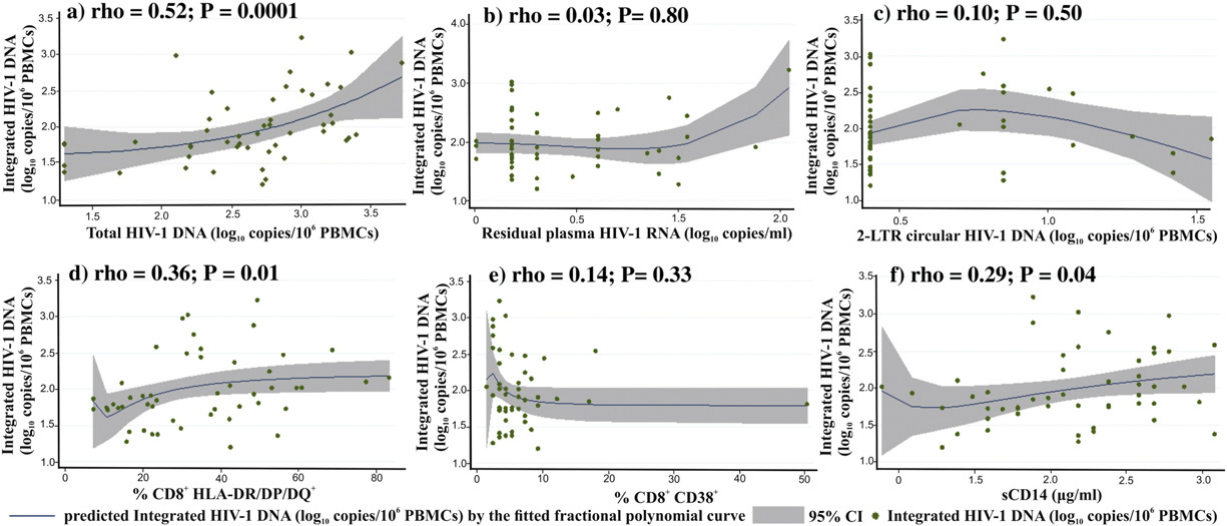
Correlation between integrated HIV-1 DNA load and a) total HIV-1 DNA load, b) residual plasma HIV-1 RNA levels, c) 2-LTR circular HIV-1 DNA levels, d) frequency of CD8^+^HLA-DR/DP/DQ^+^ cells, e) frequency of CD8^+^CD38^+^ cells, and f) levels of sCD14. Scatter plots with predicted HIV-1 DNA by the fitted fractional polynomial curves (with 95% confidence interval) and Spearman's rho (with p values) are shown.

Integrated HIV-1 DNA load was positively correlated with total HIV-1 DNA load (p < 0.0001), frequency of CD8^+^HLA-DR/DP/DQ^+^ cells (p = 0.01), and sCD14 levels (p = 0.04), but not with the levels of residual plasma HIV-1 RNA (p = 0.81) and 2-LTR circular HIV-1 DNA (p = 0.50), or the frequency of CD8^+^CD38^+^ cells (p = 0.33) (Fig. 1). The associations were also tested by univariate and multivariable linear regression analysis (Table 4). A first model was built including pre-ART viral load, duration of suppressive ART, CD4 cell counts, residual plasma HIV-1 RNA levels, frequency of CD8^+^HLA-DR/DP/DQ^+^ cells, and sCD14 levels. In this adjusted model, integrated HIV-1 DNA load was a mean of 0.5 log_10_ copies higher for each 50% increment in the frequency of CD8^+^HLA-DR/DP/DQ^+^ cells (95% CI 0.2, 0.9; p = 0.01) (Table 4).

The association between integrated HIV-1 DNA load and frequency of CD8^+^HLA-DR/DP/DQ^+^ cells was confirmed in a separate model including the nadir CD4 cell count in place of pre-ART viral load. In this second model integrated HIV-1 DNA load was mean 0.5 log_10_ copies higher for each 50% increase in the frequency of CD8^+^HLA-DR/DP/DQ^+^ cells (95% CI 0.1, 0.8; p = 0.02) (Supplementary Table 2). In a sensitivity analysis, integrated HIV-1 DNA was replaced with total HIV-1 DNA in the two multivariable models (Supplementary Table 3). In the model including pre-ART viral load as one of variables, total HIV-1 DNA load was 0.4 log_10_ copies higher for each 50% increase in the frequency of CD8^+^HLA-DR/DP/DQ^+^ cells (95% CI − 0.02, 0.8; p = 0.06); in the model including the nadir CD4 cell count in place of pre-ART viral load, total HIV-1 DNA load was similarly 0.5 log_10_ copies higher for each 50% increase in the frequency of CD8^+^HLA-DR/DP/DQ^+^ cells (95% CI − 0.1, 0.8; p = 0.12). We found no evidence that the association between integrated HIV-1 DNA load and frequency of CD8^+^HLA-DR/DP/DQ^+^ cells varied by HIV-1 subtype (B vs. non-B) (not shown).

## 4 Discussion

This analysis of subjects receiving first-line NNRTI-based ART demonstrated that integrated HIV-1 DNA load did not differ by duration of suppressive therapy and was positively associated with the frequency of CD8 cells expressing HLA-DR/DP/DQ. Subjects with higher integrated HIV-1 DNA load also had higher levels of sCD14, although the association did not persist in adjusted analyses. Whilst there was a predictable positive correlation with total HIV-1 DNA levels, integrated HIV-1 DNA load did not show a correlation with putative measures of recent HIV-1 replication (residual plasma HIV-1 RNA, 2-LTR circular HIV-1 DNA), or with the frequency of CD4 and CD8 cells expressing CD38.

Previous studies reported that during suppressive ART integrated HIV-1 DNA shows a constant load and little evidence of genetic evolution (Siliciano et al., 2003; Josefsson et al., 2013; von Stockenstrom et al., 2015; Besson et al., 2014). Our study adds to these previous analyses by demonstrating that HIV-1 DNA load did not differ by duration of suppressive therapy in a population with a relatively homogenous treatment history and exact requirements in terms of evidence of plasma viral load suppression < 50 copies/ml. There were indications that subjects treated for longer had lower levels of 2-LTR circular HIV-1 DNA and residual plasma HIV-1 RNA, accompanied by a reduction in the frequency of CD4^+^CD38^+^ and CD8^+^CD38^+^ cells, together suggesting that control of virus replication and resolution of immune dysfunction improve with longer duration of therapy. In contrast, the frequency of CD8^+^HLA-DR/DP/DQ^+^ cells was also not related to the duration of suppressive ART and we were able to quantify the association between two key parameters of virus persistence and immune activation, whereby integrated HIV-1 DNA load increased by 0.5 log_10_ copies/10^6^ PBMC for each 50% increase in the frequency of CD8^+^HLA-DR/DP/DQ^+^ cells.

The function of CD8^+^ cells expressing HLA-DR/DP/DQ^+^ remains to be fully elucidated and may include both regulatory and effector functions (Arruvito et al., 2014; Saez-Cirion et al., 2007; Zubkova et al., 2014). In the context of suppressive ART, CD8^+^CD38^−^/HLA-DR^+^ cells may be maintained by low-level expression of HIV or, other persistent pathogens, including microbial translocation from the gut. It has been proposed that CD8^+^ cells expressing HLA-DR without CD38 are preferentially generated in response to low antigenic stimulation and that by retaining good effector function, may play a role in suppressing HIV replication in elite controllers, as well as clearing hepatitis C infection (Saez-Cirion et al., 2007; Zubkova et al., 2014). It may seem therefore counterintuitive that CD8^+^HLA-DR/DP/DQ^+^ cells should have a positive (rather than inverse) correlation with integrated HIV-1 DNA load. Yet, in line with previous data (Hatano et al., 2013), our adjusted analyses showed a positive association between frequency of CD8^+^38-HLA-DR/DP/DQ^+^ cells and integrated HIV-1 DNA load. Several hypotheses may be proposed to explain the observed positive association. Firstly, low-level HIV production may both stimulate CD8^+^HLA-DR/DP/DQ^+^ cells and continuously replenish the integrated reservoir. Further, CD8^+^HLA-DR/DP/DQ^+^ cells may directly stimulate HIV-infected CD4 cells, causing their proliferation and expansion of the reservoir, which can be measured as increased integrated HIV-1 DNA load (Klatt et al., 2013). Thirdly, a stimulant or multiple stimulants may act simultaneously on CD8^+^HLA-DR/DP/DQ^+^ cells and HIV-infected CD4 cells, resulting in an indirect association between the two parameters.

The population we studied did not overall show evidence of ongoing HIV replication. Patients experienced no viral load rebound > 50 copies/ml during follow-up (Doyle et al., 2012). In line with previous studies, just over half had traces of detectable plasma HIV-1 RNA (Palmer et al., 2008), whereas a third had detectable intracellular 2-LTR circular HIV-1 DNA. There was no association however between these two putative markers of recent HIV-1 replication and either integrated HIV-1 DNA load, or the frequency of CD8^+^HLA-DR/DP/DQ^+^ cells (data not shown). Whilst this indicates that HIV production was unlikely, the finding may also reflect insufficient sensitivity of the analytic systems and the limitation of assaying peripheral blood (Cockerham et al., 2014). It will be important to determine the antigenic specificity of CD8^+^HLA-DR/DP/DQ^+^ cells, for example against persistent viruses such as CMV and EBV (Gianella et al., 2014). The two herpes viruses were not commonly detected in our population, which is consistent with containment by effective immune responses. One other aspect that warrants investigation is the relationship with levels of sCD14, which are an important predictor of disease progression and mortality in both treated and untreated patients (Sandler et al., 2011; Marchetti et al., 2011; Hunt et al., 2014). In this study, median sCD14 levels were within the range reported in healthy HIV-negative controls (Sandler et al., 2011). However, patients whose integrated HIV-1 DNA load fell within the highest quartile showed significantly higher sCD14 levels than those with integrated HIV-1 DNA load in the lowest quartile, a finding that warrants further investigation in larger cohorts.

A strong positive association was measured between total and integrated HIV-1 DNA, supporting the notion that integrated HIV-1 DNA is the most prevalent form of HIV-1 DNA during suppressive ART (Graf and O'Doherty, 2013), and total HIV-1 DNA was associated with the frequency of CD8^+^HLA-DR/DP/DQ^+^ cells. Two previous studies have reported an association between the frequency of CD8 cells expressing HLA-DR and total HIV-1 DNA load in peripheral blood (Hatano et al., 2013; Cockerham et al., 2014). One study was unable to detect a consistent association between the expression of activation markers on CD8 cells and integrated HIV-1 DNA load among 19 subjects (Cockerham et al., 2014). The reasons for the discrepant findings are unclear, and may include a smaller and more heterogeneous study population relative to this cohort, as well as possible differences in the methods to quantify integrated HIV-1 DNA load.

This study has limitations. Parameters were measured cross-sectionally, albeit after stratifying recruitment according to duration of ART, and causality of the observed associations cannot be concluded. Study size limited the number of variables included in the multivariable analysis of factors associated with integrated HIV-1 DNA load, and unmeasured variables may have contributed to the findings. Importantly, the cohort had received NNRTI-based ART exclusively, and findings may not necessarily be extrapolated to other treatment regimens. Further, we measured CD38 and HLA-DR/DP/DQ expression on CD8 cells separately. Unlike the frequency of CD8^+^HLA-DR/DP/DQ^+^ cells however, the frequency of CD8^+^CD38^+^ cells declined with duration of suppressive ART and showed no statistical association with integrated HIV-1 DNA load. Further analyses are needed to confirm the association between integrated HIV-1 DNA load and CD8^+^CD38^−^HLA-DR/DP/DQ^+^ cells, characterise the antigenic specificity of CD8^+^HLA-DR/DP/DQ^+^ cells, and determine the direction of causality. Moreover, the data describe CD8 cells expressing HLA-DR/DP/DQ and it will be of interest to study the association between integrated HIV-1 DNA load and individual HLA isotypes. Meanwhile, our data add to growing evidence indicating that a complex interplay between HIV-1 persistence and immune activation continues over many years of stably suppressive ART.

## Authors' contribution

AMG and AP designed the study. AMG managed study governance and patient recruitment. AR performed the laboratory work under AMG's supervision and with support from AB, GP, MK, WdS, and LV. MS contributed to the production and analysis of the 2-LTR circular HIV-1 DNA results under DR's supervision. ACL performed the statistical analysis. AMG and AR wrote the manuscript, which was reviewed by all authors. AMG, AB, and AR revised the manuscript with support from ACL. The Eras Study group members contributed to patient recruitment.

## Funding

The study was supported by research awards from the European AIDS Treatment Network (NEAT), the British HIV Association, the Collaborator for AIDS Research on Eradication (CARE; U19 AI096113), the UCSD CFAR (AI306214), the Department of Veterans Affairs, and the James B. Pendleton Charitable Trust. The funding sources had no role in the writing of the manuscript or the decision to submit for publication. The corresponding author (AMG) had full access to all the data in the study and had the final responsibility for the decision to submit for publication.

## Conflict of interest

The authors have no conflict of interest to declare.

## Figures and Tables

**Table 1:** Characteristics of the study population overall and stratified by duration of suppressive therapy as below (Group I) or above (Group II) the median of 6.4 years.

Characteristic	Total (n = 50)	Group I (n = 25)	Group II (n = 25)	P
Male n (%)	40 (80)	22 (88)	18 (72)	0.29
Age median years (IQR)	46 (40–53)	44 (36–49)	48 (43–55)	0.04
HIV-1 subtype B n (%)	32 (64)	18 (72)	14 (56)	0.38
Nadir CD4 count median cells/mm^3^ (IQR)	206 (110–265)	246 (160–292)	176 (97–213)	0.007
Duration of ART median years (IQR)	6.7 (3.4–9.1)	3.4 (2.5–5.0)	9.2 (8.0–10.3)	< 0.001
Duration of suppressive ART median years (IQR)^a^	6.4 (3.1–8.8)	3.1 (2.1–4.8)	9.0 (7.7–10.0)	< 0.001
Efavirenz as initial NNRTI n (%)	40 (80)	22 (44)	18 (36)	0.29
Changed NRTI backbone n (%)	25 (50)	4 (16)	21 (84)	< 0.001
Pre-ART HIV-1 RNA median log_10_ copies/ml (IQR)	5.0 (4.7–5.5)	4.8 (4.5–5.3)	5.1 (4.9–5.6)	0.09
Detectable residual plasma HIV-1 RNA n (%)	29 (58)	15 (60)	14 (56)	1.00
Residual HIV-1 RNA median copies/ml (IQR)^b^	2 (2–4)	2 (2–7)	2 (2–4)	0.33
Detectable 2-LTR circular HIV-1 DNA n (%)	16 (32)	10 (40)	6 (25)	0.36
2-LTR circular HIV-1 DNA median copies/10^6^ PBMC (IQR)^c^	3 (3–7)	3 (3–7)	3 (3–5)	0.28
Total HIV-1 DNA median log_10_ copies/10^6^ PBMC (IQR)	2.6 (2.3–2.9)	2.6 (2.4–2.9)	2.5 (2.2–2.7)	0.36
Integrated HIV-1 DNA median log_10_ copies/10^6^ PBMC (IQR)	1.9 (1.7–2.2)	1.9 (1.7–2.1)	1.8 (1.7–2.4)	0.91
CD4 count median cells/mm^3^ (IQR)	572 (478–734)	552 (442–606)	640 (502–794)	0.05
CD4^+^CD26^+^ median percentage (IQR)	54 (43–63)	54 (42–63)	54 (44–63)	0.55
CD4^+^ CD38^+^ median percentage (IQR)	22 (17–34)	25 (21–35)	20 (14–24)	0.02
CD4^+^CD69^+^ median percentage (IQR)	2 (1–2)	1 (1–3)	2 (1–2)	0.48
CD8^+^CD38^+^ median percentage (IQR)	5 (3–7)	6 (3–8)	4 (3–6)	0.04
CD8^+^HLA-DR/DP/DQ^+^ median percentage (IQR)	32 (20–48)	37 (22–53)	30 (16–42)	0.19
sCD14 median μg/ml (IQR)	2.2 (1.8–2.6)	2.0 (1.6–2.6)	2.2 (2.0–2.7)	0.16

IQR = interquartile range; ART = antiretroviral therapy; NRTI = nucleoside/nucleotide reverse transcriptase inhibitor; NNRTI = non-nucleoside reverse transcriptase inhibitor; PBMC = peripheral blood mononuclear cells; sCD14 = soluble CD14.

a Defined as the length of time following the first viral load < 50 copies/ml; it ranged between 0.7 and 6.4 years in Group 1, and between 6.4 and 14.4 years in Group II.

b Samples with undetectable HIV-1 RNA were assigned an arbitrary value of 1.5 copies/ml.

c Samples with undetectable 2-LTR circular HIV-1 DNA were assigned an arbitrary value of 2.5 copies/10^6^ PBMC.

**Table 2:** Univariate linear regression analysis of mean difference in log-transformed virological and immunological variables per 10 years of suppressive antiretroviral therapy.

Variable^a^	Mean difference	95% CI	P
Residual HIV-1 RNA copies/ml	− 0.27	− 0.57, 0.03	0.08
2-LTR circular HIV-1 DNA copies/10^6^ PBMC	− 0.34	− 0.59, − 0.08	0.01
Total HIV-1 DNA copies/10^6^ PBMC	− 0.12	− 0.56, 0.32	0.60
Integrated HIV-1 DNA copies/10^6^ PBMC	0.22	− 0.19, 0.62	0.28
CD4 count cells/mm^3^	0.14	0.00, 0.27	0.05
CD4^+^CD26^+^ percentage	− 0.03	− 0.02, 0.10	0.69
CD4^+^CD38^+^ percentage	− 0.23	− 0.39, − 0.07	0.01
CD4^+^CD69^+^ percentage	− 0.11	− 0.28, 0.07	0.22
CD8^+^CD38^+^ percentage	− 0.30	− 0.54, − 0.07	0.01
CD8^+^HLA-DR/DP/DQ^+^ percentage	− 0.15	− 0.37, 0.07	0.17
sCD14 μg/ml	0.07	− 0.03, 0.16	0.19

CI = Confidence interval; PBMC = peripheral blood mononuclear cells; sCD14 = soluble CD14.

a Variables in log_10_.

**Table 3:** Comparative analysis of subjects whose integrated HIV-1 DNA load fell within the lowest or the highest quartiles^a^.

Characteristic	Lowest quartile (n = 13)	Highest quartile (n = 13)	P
Integrated HIV-1 DNA median log_10_ copies/10^6^ PBMC (IQR)	1.4 (1.4–1.6)	2.6 (2.5–2.9)	< 0.0001
Male n (%)	9 (70)	11 (84)	0.64
Age median years (IQR)	40 (31–49)	47 (43–53)	0.07
HIV-1 subtype B n (%)	8 (61)	5 (38)	0.43
Nadir CD4 count median cells/mm^3^ (IQR)	180 (80–240)	208 (118–260)	0.41
Duration of ART median years (IQR)	6.7 (5.7–8.2)	9.1 (3.0–10.7)	0.29
Duration of suppressive ART median years (IQR)	6.6 (5.7–7.7)	8.9 (2.7–10.6)	0.24
Efavirenz as initial NNRTI n (%)	13 (100)	10 (77)	0.22
Changed NRTI backbone n (%)	8 (61)	5 (38)	0.43
Pre-ART HIV-1 RNA median log_10_ copies/ml (IQR)	4.9 (4.6–5.5)	5.1 (5.0–5.5)	0.26
Detectable residual plasma HIV-1 RNA n (%)	9 (69)	6 (46)	0.43
Residual HIV-1 RNA median copies/ml (IQR)	2 (2–3)	2 (2–5)	0.89
Detectable 2-LTR circular HIV-1 DNA n (%)	4 (31)	6 (46)	0.68
2-LTR circular HIV-1 DNA median copies/10^6^ PBMC (IQR)	3 (3–7)	3 (3–7)	0.65
Total HIV-1 DNA median log_10_ copies/10^6^ PBMC (IQR)	2.4 (2.1–2.6)	2.8 (2.7–3.0)	0.003
CD4 count median cells/mm^3^ (IQR)	721 (552–758)	513 (482–640)	0.25
CD4^+^CD26^+^ median percentage (IQR)	53 (34–60)	54 (44–59)	0.85
CD4^+^ CD38^+^ median percentage (IQR)	23 (17–34)	20 (16–24)	0.28
CD4^+^CD69^+^ median percentage (IQR)	2 (1–2)	2 (1–2)	0.64
CD8^+^CD38^+^ median percentage (IQR)	4 (3–7)	3 (2–7)	0.43
CD8^+^HLA-DR/DP/DQ^+^ median percentage (IQR)	24 (16–37)	35 (32–49)	0.01
sCD14 median μg/ml (IQR)	1.8 (1.6–2.3)	2.4 (2.1–2.7)	0.04

PBMC = peripheral blood mononuclear cells; IQR = interquartile range; ART = antiretroviral therapy; NNRTI = non-nucleoside reverse transcriptase inhibitor; sCD14 = soluble CD14.

a Quartile cut-offs = 1.7 and 2.2 log_10_ copies/10^6^ PBMC.

**Table 4:** Univariate and multivariable linear regression analysis of factors associated with the mean difference in integrated HIV-1 DNA load^a^.

	Univariate			Multivariable		
Factor	Mean difference	95% CI	P	Mean difference	95% CI	P
Age, per 10 years higher	0.11	− 0.03, 0.25	0.11			
HIV-1 subtype B vs. non B	0.11	− 0.18, 0.39	0.46			
Nadir CD4 count per 100 cell/mm^3^ higher	0.06	− 0.04, 0.17	0.25			
Duration of suppressive ART per 10 years longer	0.22	− 0.19, 0.62	0.28	0.23	− 0.20, 0.66	0.30
Changed NRTI backbone yes vs. no	− 0.14	− 0.41, 0.13	0.30			
Pre-ART HIV-1 RNA per log_10_ copies/ml higher	0.10	− 0.08, 0.28	0.27	0.13	− 0.05, 0.30	0.15
Residual HIV-1 RNA per log_10_ copies/ml higher	0.20	− 0.17, 0.57	0.28	0.27	− 0.08, 0.62	0.13
2-LTR circular HIV-1 DNA per log_10_ copies/10^6^ PBMC higher	0.04	− 0.39, 0.46	0.85			
CD4 count per 100 cells/mm^3^ higher	0.03	− 0.02, 0.08	0.25	0.02	− 0.03, 0.07	0.38
CD4^+^CD26^+^ per 50% higher	0.01	− 0.44, 0.47	0.96			
CD4^+^CD38^+^ per 50% higher	− 0.78	− 2.11, 0.54	0.24			
CD4^+^CD69^+^ per 50% higher	1.02	− 5.29, 7.33	0.75			
CD8^+^CD38^+^ per 50% higher	− 0.27	− 1.22, 0.67	0.57			
CD8^+^HLA-DR/DP/DQ^+^ per 50% higher	0.38	0.02, 0.74	0.04	0.51	0.15, 0.86	0.01
sCD14 per log_10_ μg/ml higher	0.97	− 0.14, 2.08	0.09	0.90	− 0.16, 1.97	0.10

PBMC = peripheral blood mononuclear cells; ART = antiretroviral therapy; NRTI = nucleoside/nucleotide reverse transcriptase inhibitor; sCD14 = soluble CD14.

a Mean difference in log_10_ copies/10^6^ PBMC.
