# Supplementary material for: During Stably Suppressive Antiretroviral Therapy Integrated HIV-1 DNA Load in Peripheral Blood is Associated with the Frequency of CD8 Cells Expressing HLA-DR/DP/DQ
Source: eBioMedicine. 2015 Jul 21;2(9):1153–9. doi: 10.1016/j.ebiom.2015.07.025 (PMC4588402; doi:10.1016/j.ebiom.2015.07.025)
Supplement: Supplementary file 1 — Supplementary tables. [file mmc1.docx]

**Supplementary Table 1.** Primers used in the integrated HIV-1 DNA assay

|  | **Target** | **Primer sequence (5’ – 3’)** |
| --- | --- | --- |
| PCR 1 | Human Alu (sense) | GCCTCCCAAAGTGCTGGGATTACAG |
|  | HIV-1 gag (anti-sense) | GTTCCTGCTATGTCACTTCC |
| PCR2 | HIV-1 RU5 (sense) | TTAAGCCTCAATAAAGCTTGCC |
|  | HIV-1 RU5 (anti-sense) | GTTCGGGCGCCACTGCTAGA |
|  | HIV-1 RU5 (probe) | FAM/CCAGAGTCA/ZEN/CACAACAGACGGGCACA/3IABKFQ/ |

**Supplementary Table 2.** Multivariable linear regression analysis of factors associated with the mean difference in integrated HIV -1 DNA load over 10 years of suppressive antiretroviral therapy in a model including nadir CD4 cell count

| **Factor** | **Mean difference^a^** | **95% CI** | **P** |
| --- | --- | --- | --- |
| Nadir CD4 count per 100 cell/mm^3^ higher | 0.04 | -0.09, 0.16 | 0.55 |
| CD4 count per 100 cells/mm^3^ higher | 0.01 | -0.04, 0.06 | 0.60 |
| Duration of suppressive ART per 10 years longer | 0.37 | -0.12, 0.86 | 0.13 |
| Residual HIV-1 RNA per log_10_ copies/ml higher | 0.26 | -0.10, 0.62 | 0.15 |
| CD8^+^HLA-DR/DP/DQ^+^ per 50% higher | 0.45 | 0.08, 0.82 | 0.02 |
| sCD14 per log_10_ μg/ml higher | 0.76 | -0.40, 1.92 | 0.19 |

^a^Mean difference in integrated HIV-1 DNA in log_10_ copies/10^6^ PBMC. PBMC= peripheral blood mononuclear cells; ART= antiretroviral therapy.

**Supplementary Table 3.** Sensitivity analysis replacing integrated with total HIV-1 DNA in the two multivariable models^a^.

| **Factor** | **Model 1** | | | | | **Model 2** | | |
| --- | --- | --- | --- | --- | --- | --- | --- | --- |
|  | **Mean difference** | | **95% CI** | **P** | **Mean difference** | | **95% CI** | **P** |
| Nadir CD4 count per 100 cell/mm^3^ higher | | - | - | - | -0.02 | | -0.16, 0.13 | 0.83 |
| CD4 count per 100 cells/mm^3^ higher | | -0.02 | -0.08, 0.04 | 0.53 | -0.02 | | -0.09, 0.04 | 0.43 |
| Duration of suppressive ART per 10 years longer | | -0.12 | -0.62, 0.39 | 0.65 | -0.01 | | -0.60, 0.58 | 0.98 |
| Pre-ART HIV-1 RNA per log_10_copies/ml higher | | 0.23 | 0.03, 0.43 | 0.03 | - | | - | - |
| Residual HIV-1 RNA per log_10_ copies/ml | | 0.14 | -0.26, 0.55 | 0.48 | 0.14 | | -0.30, 0.57 | 0.53 |
| CD8^+^HLA-DR/DP/DQ^+^ per 50% higher | | 0.39 | -0.02, 0.80 | 0.06 | 0.35 | | -0.09, 0.80 | 0.12 |
| sCD14 per log_10_ μg/ml higher | | 0.56 | -0.68, 1.78 | 0.37 | 0.57 | | -0.83, 1.97 | 0.41 |

^a^Mean difference in log_10_ copies/10^6^ PBMC. Model 1 included pre-ART viral load; model 2 replaced pre-ART viral load with nadir CD4 cell count. PBMC: peripheral blood mononuclear cells; ART: antiretroviral therapy.
